# Predictability of Insulin Resistance Based on the Clinical Manifestations Among Male Medical Students of a Private College in Saudi Arabia

**DOI:** 10.7759/cureus.60327

**Published:** 2024-05-15

**Authors:** Yousria A Badawy, Ahmed H Almakrami, Abdullah J Alata, Emad Z Abujamea, Louai M Khaliifa

**Affiliations:** 1 Family Medicine, Ibn Sina National College for Medical Studies, Jeddah, SAU; 2 Medicine and Surgery, Ibn Sina National College for Medical Studies, Jeddah, SAU; 3 Pharmacy, Ibn Sina National College for Medical Studies, Jeddah, SAU

**Keywords:** psoriasis, androgenic alopecia, acanthosis nigricans, hidradenitis suppurativa, insulin resistance, carb cravings, dermatological manifestations, day sleep, risk factors, waist circumference

## Abstract

Background

Numerous clinical signs and symptoms are thought to be associated with insulin resistance. The purpose of this study was to examine the prevalence of insulin resistance among male medical students attending a private Saudi Arabian institution, based on clinical indications.

Methods

A convenient non-probability sample consisting of 241 male medical students was used to conduct cross-sectional research. Each participant had an in-person interview as well as anthropometric measurements. The interview consisted of a questionnaire that was used to assess demographic data and clinical manifestations related to insulin resistance.

Results

The study demonstrated the connection between a few dermatological symptoms and waist circumference as an indicator of insulin resistance. In both the high and normal waist circumference groups, acne was the most common symptom. There was no correlation found between waist circumference and psoriasis, hidradenitis suppurativa, androgenic alopecia, alopecia areata, or vitiligo. Nevertheless, as an indicator of insulin resistance, waist circumference was statistically significantly correlated with both skin tags and acanthosis nigricans. Most students had excessive day sleep, foggy brains, struggled with planning and solving problems, and had a memory that became worse in the past few years. In addition, many students feel hungry even after eating some sweets and usually have extreme thirst.

Conclusion

Among medical students, skin tags, acanthosis nigricans, and acne were the most prevalent dermatological manifestations. Clinicians need to be aware that skin conditions, sleep difficulties throughout the day, changes in cognition, and food cravings might all be indicators of internal changes and/or illnesses such as diabetes and prediabetes.

## Introduction

One significant pathogenic component of numerous metabolic diseases, such as prediabetes, is insulin resistance. There is not a simple clinical test to determine a person's level of insulin resistance. Previous investigators evaluated the predictive power of various anthropometric measures (weight, height, and waist circumference) and biochemical indicators that are utilized in clinical practice to determine insulin sensitivity [1].

A study among healthy Omani medical students was conducted regarding insulin resistance and stated that there is a high prevalence of insulin resistance (16%). In that study, it was clear that there was a high prevalence of obesity (26%) among the sample [2]. Furthermore, a Saudi Arabian study from 2018 found a connection between insulin resistance and dermatological symptoms. An analysis was conducted on skin complaints from medical students. Hair loss was the most prevalent skin complaint, followed by acne and pimples, as well as greasy, waxy, and flaky scalp areas. Skin tags and warts were the least common complaints. A great majority of students have reported darker patches or acanthosis nigricans around different areas in their bodies [3].

A recent study in Riyadh has documented a high prevalence of stress and poor sleep quality in a sample of Saudi medical school students, and the sample had shown signs of tiredness and feeling sleepy after waking up, and since sleep quality and quantity play a significant role in the cognitive function, it was obvious that student who had less sleep hours had some cognitive impairments in the form of lack of concentration or the inability to make quick decisions [4]. Another study that was conducted in Riyadh among medical students focused on daytime naps. It was shown that daytime nap was associated with poor sleep quality, which is prevalent among the sample, and it may be a sign of insulin resistance if it is combined with other risks and clinical manifestations of insulin resistance, which would need lifestyle modifications and reflection on the causes of these conditions [5].

In a study that measured the significance of stress and burnout on medical students, it was indicated that specific lifestyle and behavioral factors, including sweet food consumption and cravings, were associated with medical students’ daily habits, which are major predisposing factors as well as a clinical sign of insulin resistance [6].

Throughout the literature [2,3], there is consistent evidence that insulin resistance is influenced by many factors, some of which are significant among medical students without proper documentation. The collection of the common clinical manifestations that may predict insulin resistance among medical students in Jeddah, and their prevalence will be key concepts to this research.

## Materials and methods

Study design and setting

The research design used in the study by Alghamdi et al. (2023) [7] on male Ibn Sina medical students is replicated in this work. Our study, in contrast, aims to determine the prevalence of clinical manifestation linked to insulin resistance and concentrates on male medical students from the same college. The study was conducted in a Saudi Arabian private college of medical sciences.

Sampling technique

A convenient nonprobability sampling method was applied. Using Epi Info version 7 (Centers for Disease Control and Prevention, Atlanta, GA), with a 95% confidence interval and 5% margin of error, the sample size was calculated for a single population based on an estimated prevalence of 23% of insulin resistance, as reported by a prior study in a comparable cohort [8]. The sample size was 241.

Data collection

Every student had an interview consisting of a questionnaire that was used to assess demographic data and clinical manifestation related to insulin resistance as well as anthropometric measurement. For clinical manifestation, data about dermatological changes, cognitive affection, carbohydrate cravings, and the presence or absence of day sleep were assessed. For day sleep, the Epworth Sleepiness Scale (ESS) was used [9]. The anthropometric data included waist circumference, weight, and height. For waist circumference, the cut-off point of 102 cm or more was used as an indication of high waist circumference [7].

Data analysis

Microsoft Excel (Microsoft Corporation, Redmond, WA) was used to gather and organize data. SPSS version 22 (IBM Corp., Armonk, NY) was used to analyze the data. For both qualitative and quantitative data, percentages and frequencies were computed. A p-value of less than 0.05 was regarded as statistically significant when using the chi-square test.

Ethical consideration

Approval for this study was obtained from the Ibn Sina National College for Medical Studies (approval number: IRRB-01-28022022). All information obtained was kept strictly confidential. The data collection sheet included a consent form for participation.

## Results

Table 1 presents the clinical and sociodemographic information of male medical students attending a Saudi Arabian private medical institution. In all, 241 medical students took part in the research. Out of the total participants, less than half (41.9%) belonged to the 20-22 age group. Conversely, a small percentage (8.7%) belonged to the 25-28 age bracket. Although the study's participants included students from all grade levels, a large proportion were in their first and fourth years (22.8% and 20.3%, respectively). But the least involved were sixth-year students (11.2%). Of those with a body mass index (BMI) calculation, 52.7% were obese or overweight. A third (19.5%) of them had a big waist circumference. A quarter (25.3%) of students were smokers.

**Table 1:** Sociodemographic and status clinical profile among male medical students of a private medical college in Saudi Arabia (2022; n = 241)

Variable	Frequency	Percent
Age categories in years		
Less than 20	29	12.0
20-22	101	41.9
23-24	90	37.3
25-28	21	8.7
Level of education		
First	55	22.8
Second	43	17.8
Third	36	14.9
Fourth	49	20.3
Fifth	31	12.9
Sixth	27	11.2
BMI categories		
Normal weight	114	47.3
Overweight or obese	127	52.7
Waist circumference		
Normal waist circumference	194	80.5
High waist circumference	47	19.5
Smoking		
Never	141	58.5
Former	39	16.2
Current	61	25.3
Total	241	100.0

Most of the students in the sample were medically free, as shown in Table 2; however, a percentage of 4.1% were reported to have diabetes mellitus, followed by 2.9% had dyslipidemia and 2.5% had hypertension. Additionally, a minority (2.5%) of the students were revealed to have a hormonal disorder or taking corticosteroids (2.1%).

**Table 2:** Health status data among male medical students of a private medical college in Saudi Arabia (2022; n = 241)

Variables	Frequency	Percentage
Dyslipidemia		
Yes	7	2.9
No	234	97.1
Diabetes mellitus		
Yes	10	4.1
No	231	95.9
Hypertension		
Yes	6	2.5
No	235	97.5
Hormonal disorders		
Yes	6	2.5
No	235	97.5
On corticosteroids		
Yes	5	2.1
No	236	97.9

The results of the dermatologic manifestations among male medical students are presented in Table 3. Almost a quarter (22.4%) of the students had acne, with slightly over one-tenth having skin tags (11.6%), and less than one-tenth (7.9%) suffered from acanthosis nigricans. As regards androgenic alopecia, exactly 5% mentioned that they suffered from it. On the other hand, only 2% had alopecia areata. Considering psoriasis, a minority (1.2%) revealed that they had it somewhere in their bodies. Also, a tiny minority had hidradenitis suppurativa (0.8%) and vitiligo (0.8%).

**Table 3:** Prevalence of dermatological manifestations common with insulin resistance among male medical students (n = 241)

Dermatological manifestations	Frequency	Percentage
Acne		
Yes	54	22.4
No	187	77.6
Total	241	100.0
Skin tag		
Yes	28	11.6
No	213	88.4
Total	241	100.0
Acanthosis nigricans		
Yes	19	7.9
No	222	92.1
Total	241	100.0
Androgenic alopecia		
Yes	12	5.0
No	229	95.0
Total	241	100.0
Alopecia areata		
Yes	5	2.1
No	236	97.9
Total	241	100.0
Psoriasis		
Yes	3	1.2
No	238	98.8
Total	241	100.0
Hidradenitis suppurativa		
Yes	2	0.8
No	239	99.2
Total	241	100.0
Vitiligo		
Yes	2	0.8
No	239	99.2
Total	241	100.0

The results of the current study presented in Table 4 show the association of some dermatological manifestations associated with insulin resistance and waist circumference as an indicator of insulin resistance using the chi-square test. Of all the dermatological manifestations, acne was reported as the highest in both groups (27.7% and 22.1%) in the high and normal waist circumference groups, respectively, but did not demonstrate statistical significance with waist circumference. As to psoriasis, hidradenitis suppurativa, androgenic alopecia, alopecia areata, and vitiligo were not associated with waist circumference. However, acanthosis nigricans (p = 0.010) and skin tags (p = 0.005) were statistically significant in relation to waist circumference as an indicator of insulin resistance.

**Table 4:** Association of some clinical manifestations known to be associated with insulin resistance and waist circumference as an indicator of insulin resistance among male medical students of a private medical college in Saudi Arabia ^a^ Chi-square was used. * P-value was significant.

P-value^a^	High waist circumference (47), N (%)	Normal waist circumference (194), N (%)	Dermatological manifestation
Acne			
0.336	13 (27.7%)	41 (21.1%)	Yes
	34 (72.3%)	153 (78.9%)	No
Acanthosis nigricans			
0.010*	8 (17%)	11 (5.7%)	Yes
	39 (83%)	183 (94.3%)	No
Skin tags			
0.005*	11 (22.4%)	17 (8.8%)	Yes
	36 (77.6%)	177 (91.2%)	No
Androgenic alopecia			
0.622	3 (6.4%)	9 (4.6%)	Yes
	44 (93.6%)	185 (95.4%)	No
Alopecia areata			
0.977	1 (2.1%)	4 (2%)	Yes
	46 (97.9%)	190 (98%)	No
Psoriasis			
0.391	0 (0.0%)	3 (1.5%)	Yes
	47 (100%)	191 (98.5%)	No
Hidradenitis suppurative			
0.485	0 (0.0%)	2 (1%)	Yes
	47 (100%)	192 (99%)	No
Vitiligo			
0.485	0 (0.0%)	2 (1%)	Yes
	47 (100%)	192 (99%)	No
	47 (19.5%)	194 (80.5%)	Total, n (%)

Table 5 shows the relation between waist circumference as an indicator of insulin resistance and some clinical manifestations known to be associated with insulin resistance. Almost three-quarters (74.7%) of male medical students had excessive day sleep. On the other hand, a minority (11.6%) of the students had foggy brains and those who had difficulty concentrating accounted for 16.2%. Considering struggling with planning and solving problems, there were 15.8% of the total number of students. Those who have a memory that became worse in the past few years were around 17% and those who have difficulty in understanding written or verbal information were only 5.8%. Those who feel hungry even after eating some sweets were around one-fourth (22.7%), but those who usually had extreme thirst or hunger were 22.2%. As to the cognitive changes, such as a foggy brain, difficulty concentrating, struggles with planning and problem-solving, worsening memory, or difficulty understanding, none were found to have a statistically significant correlation with waist circumference. Additionally, carbohydrate cravings, such as craving sweets after consuming something sweet, hunger after eating, and extreme thirst or hunger, were not statistically significant for those of high or normal waist circumference. When asked for day sleep, more than three-quarters (76.3%) of those with normal waist circumference declared that they suffer from excessive day sleep. Moreover, more than two-thirds (68.1%) of those with high waist circumference complained of the same symptom, which was also not significant.

**Table 5:** Prevalence of some clinical manifestations known to be associated with insulin resistance among male medical students of a private college in Saudi Arabia ^a^ Chi-square was used.

Waist circumference				Clinical manifestations	
P-value^a^	Total (241), n (%)	High (47), n (%)	Normal (194), n (%)		
Day sleep					
0.246	61 (25.3%)	15 (31.9%)	46 (23.7%)	Unlikely day sleep	
	180 (74.7%)	32 (68.1%)	148 (76.3%)	Excessive day sleep	
Cognitive changes - foggy brain					
0.435	28 (11.6%)	7 (14.9%)	21 (10.8%)	Yes	
	213 (88.4%)	40 (85.1%)	173 (89.2%)	No	
Difficulty concentrating					
0.862	39 (16.2%)	8 (17%)	31 (16%)	Yes	
	202 (83.8%)	39 (83%)	163 (84%)	No	
Struggling with planning					
0.282	38 (15.8%)	5 (10.6%)	33 (17%)	Yes	
	203 (84.2%)	42 (89.4%)	161 (83%)	No	
Memory became worse					
0.999	41 (17%)	8 (17%)	33 (17%)	Yes	
	200 (83%)	39 (83%)	161 (83%)	No	
Difficulty understanding					
0.851	14 (5.8%)	3 (6.4%)	11 (5.7%)	Yes	
	227 (94.2%)	44 (96.6%)	183 (94.3%)	No	
Food cravings - crave sweat after eating					
0.102	69 (28.6%)	18 (38.3%)	51 (26.3%)	Yes	
	172 (71.4%)	29 (61.7%)	143 (73.7%)	No	
Hungary after eating					
0.224	51 (21.2%)	13 (27.7%)	38 (19.6%)	Yes	
	190 (87.7%)	34 (72.3%)	156 (80.4%)	No	
Extreme thirst or hunger					
0.246	51 (21.2%)	13 (27.7%)	38 (19.6%)	Yes	
	190 (87.7%)	34 (72.3%)	156 (80.4%)	No	
	241 (100%)	47 (19.5%)	194 (80.5%)		Total, n (%)

## Discussion

As regards the prevalence of dermatological manifestation reported in the present study as well as its relation to waist circumference as an indicator of insulin resistance, acne was the most common dermatological manifestation in the current study. Acne vulgaris is a chronic inflammatory skin disease strongly associated with insulin resistance and both disorders share the same signal transduction pathway [10]. Acne was not statistically significant for those of normal and high waist circumference. A study provided evidence that young males affected with acne had a high body mass index and exhibited insulin resistance [11]. However, another study did not suggest a major rule of insulin resistance in acne [12]. As was noted in the assessment of the literature, several investigations revealed that acne was frequently associated with systemic disorders, most of which were connected to insulin resistance [13].

Recent studies have shown that hyperglycemic carbohydrates and insulinotropic milk/dairy products are linked to diabetes and may drive acne pathogenesis, promoting increased insulin and increased body mass index [14]. As central obesity is a major component of metabolic syndrome, it is not surprising that acne patients may frequently exhibit increased levels of serum glucose and insulin as well as insulin resistance. In this context, Western diet and lifestyle, two main actors of Western civilization, appear to be the linking points between acne, insulin resistance, and metabolic syndrome [15].

Skin tags are frequently seen in association with acanthosis nigricans, and even the present research showed very similar percentages. They were found to be statistically significant among those with high waist circumference compared to those with normal waist circumference. Although skin tags are painless and generally do not hurt, they are uncomfortable. A study reported that skin tags were observed in 77% of 109 participants with a mean body mass index of 39.6 ± 8 kg/m2, which suggests that skin tags should be considered clinical markers of hyperinsulinemia in non-diabetic obese patients [16]. A study suggested that routine assessment for acanthosis nigricans is a non-invasive and cost-effective way to identify asymptomatic overweight adolescents who are at high risk of developing insulin resistance [17].

Even though the current study found that 5% of men had androgenic alopecia and 2.1% had alopecia areata, previous studies showed that men with early onset of androgenetic alopecia (35 years) had a significantly higher risk of insulin resistance-associated disorders like obesity, hypertension, and dyslipidemia [18]. This supports the idea that early alopecia could be a clinical marker of insulin resistance. In the present study, androgenic alopecia and alopecia were not statistically significant and were more common in participants with normal waist circumference; despite that, it was shown that insulin favors vasocontraction and nutritional deficiency in hair follicles and enhances the effect of dihydrotestosterone (DTH) on follicular miniaturization resulting in alopecia [19].

The present study did not show a statistically significant association between vitiligo and high waist circumference even though a study mentioned that participants with vitiligo were more insulin-resistant compared to healthy control subjects, which supports that vitiligo may be a prediabetic condition [20]. Manifestations of psoriasis, which had an obvious association with insulin resistance [21] were not found to have a statistically significant correlation with waist circumference in the present study, despite both vitiligo and psoriasis being more prevalent in the normal waist circumference category.

A study revealed an increased frequency of insulin resistance with hidradenitis suppurative patients, which suggests hidradenitis suppurative patients should be evaluated for insulin resistance and managed accordingly [22]. It was discovered that there was a correlation between body mass index and hidradenitis suppurativa [23]. Moreover, the prevalence of hidradenitis suppurativa was higher in the obese population [24]. However, the present study did not find a correlation that was statistically significant between hidradenitis suppurative and waist circumference.

Also, the current study reported the relation between waist circumference as an indicator of insulin resistance and some clinical manifestations known to be associated with insulin resistance. Regarding day sleep, cognitive changes, and food cravings, none of these variables were found to be statistically significantly different between those of normal and high waist circumference in the present study. Moreover, participants reporting having these manifestations were more in those with normal waist circumference rather than those with high waist circumference.

The high number of participants who reported excessive day sleep could be explained by the length of classes and home studying as all participants are medical students. Also, those reported worse memory could be explained by referring to the huge load of information delivered during the medical study, which cannot be recalled in some situations, which is well understood in the medical field. Moreover, excessive hunger and thirst may be explained by only drinking or eating when reaching the feeling of extreme thirst or hunger as most of the participants’ time is spent attending classes. These results were supported by Youssef et al. (2014) [25].

## Conclusions

Since treating insulin resistance is crucial to controlling many chronic non-communicable diseases, the current study discovered that medical students had clinical manifestations of insulin resistance as well as central obesity, as indicated by high waist circumference. Among medical students, skin tags, acanthosis nigricans, and acne were the most prevalent dermatological manifestations. Clinicians need to be aware that skin conditions, sleep difficulties throughout the day, changes in cognition, and food cravings might all be indicators of internal changes and/or illnesses.
